# 902. Incidence of Influenza-Related Medical Encounters Among Adults in The United States During the 2015-2020 Influenza Seasons

**DOI:** 10.1093/ofid/ofad500.947

**Published:** 2023-11-27

**Authors:** Ian McGovern, Katherine Cappell, Alina Bogdanov, Mendel Haag

**Affiliations:** CSL Seqirus, Waltham, Massachusetts; Veradigm, Chicago, Illinois; Veradigm, Chicago, Illinois; CSL Seqirus, Waltham, Massachusetts

## Abstract

**Background:**

Research on the burden of influenza often focuses on the most impacted groups; children and older adults (65 years of age and older). This study aimed to evaluate how age impacted incidence of influenza-related outpatient visits, emergency room (ER) visits, and hospital admissions by broader adult age groups (18-49, 50-64, and 65 years and older) as well as within more granular 5-year age increments.

**Methods:**

Patients 18 years of age or older in the United States were evaluated retrospectively in five seasonal cohorts (2015-2016 through 2019-2020 seasons). Patient-level electronic medical records linked to pharmacy and medical claims were used to ascertain outcomes. Influenza-related medical encounters were identified based on influenza-related diagnostic codes (ICD-10 codes J09*–J11*). Incidence was evaluated among the overall population as well as among people with no (low-risk) and 1 or more (high-risk) risk-factors for severe outcomes following influenza infection.

**Results:**

Between 887,260 and 3,628,168 patients were included per season. When evaluated in 5-year increments, influenza-related outpatient visit incidence was highest among people 18-35 years old, and then declined with increasing age (figure 1). For ER visits, incidence tended to be elevated for patients 18-34 and then stayed somewhat flat from 35 (or 40) through 60 years of age and increased quickly after 60. Hospital admission incidence stayed somewhat flat until around 50 years of age and then increased with age. The prevalence of risk factors increased with age, with the number of patients with 1+ risk factors increasing from 38% for 18-49, 62% for 50-64, and 80% for 50-64. Across outcomes, high-risk patients consistently had higher incidence than low-risk patients, but differences were smaller than the differences in incidence between age groups. Although similar trends were observed in the broader age groups (figure 2), the 5-year age increments provided more granular insights into the effects of age on incidence of influenza.

**Figure:**
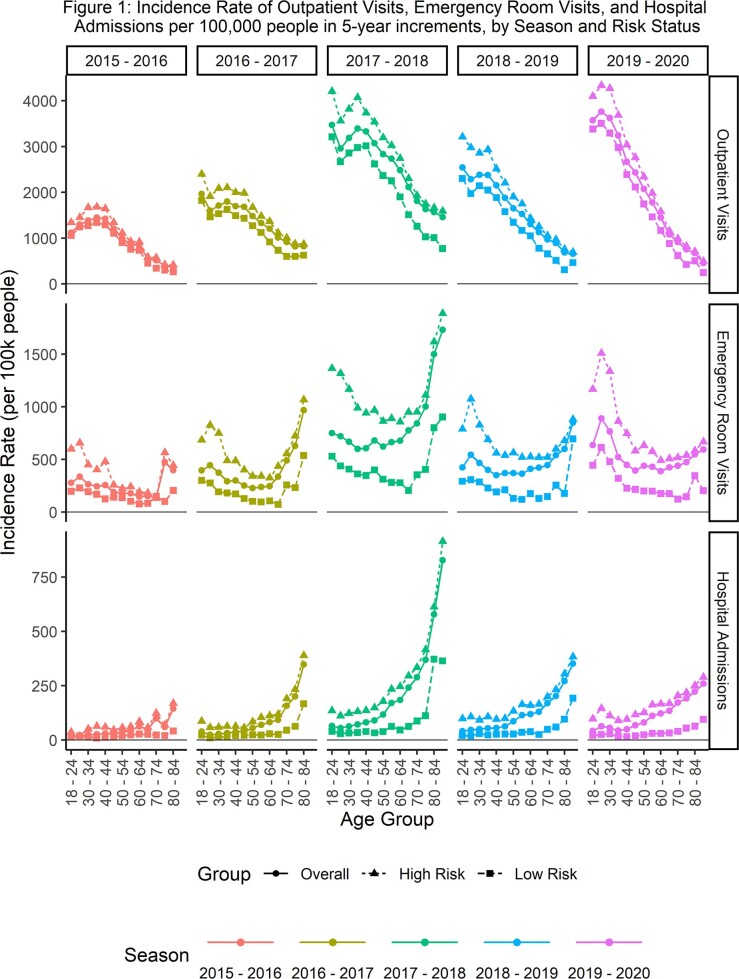

**Figure:**
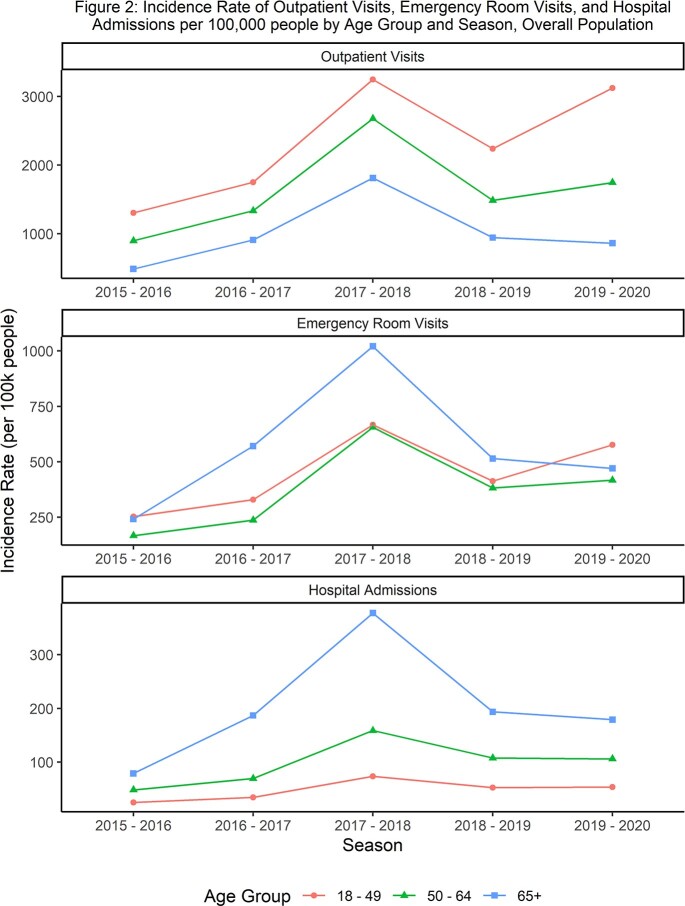

**Conclusion:**

This analysis demonstrates that changes in influenza incidence often occur gradually as patients age increases. For the most severe outcomes (hospitalizations), the incidence begins to increase around 50 years of age rather than the more common cutoff of 65 years of age.

**Disclosures:**

**Ian McGovern, MPH**, CSL Seqirus: Employment|CSL Seqirus: Stocks/Bonds **Katherine Cappell, PhD**, CSL Seqirus: Advisor/Consultant **Alina Bogdanov, MA**, Moderna, Inc.: Advisor/Consultant|Veradigm: Salary **Mendel Haag, PhD, PharmD**, CSL Seqirus: Stocks/Bonds

